# Organization and Biology of the Porcine Serum Amyloid A (SAA) Gene Cluster: Isoform Specific Responses to Bacterial Infection

**DOI:** 10.1371/journal.pone.0076695

**Published:** 2013-10-11

**Authors:** Helle G. Olsen, Kerstin Skovgaard, Ole L. Nielsen, Páll S. Leifsson, Henrik E. Jensen, Tine Iburg, Peter M. H. Heegaard

**Affiliations:** 1 Department of Veterinary Disease Biology, Faculty of Health and Medical Sciences, University of Copenhagen, Frederiksberg, Denmark; 2 Innate Immunology Group, National Veterinary Institute, Technical University of Denmark, Frederiksberg, Denmark; Temple University, United States of America

## Abstract

Serum amyloid A (SAA) is a prominent acute phase protein. Although its biological functions are debated, the wide species distribution of highly homologous SAA proteins and their uniform behavior in response to injury or inflammation in itself suggests a significant role for this protein. The pig is increasingly being used as a model for the study of inflammatory reactions, yet only little is known about how specific SAA genes are regulated in the pig during acute phase responses and other responses induced by pro-inflammatory host mediators. We designed SAA gene specific primers and quantified the gene expression of porcine SAA1, SAA2, SAA3, and SAA4 by reverse transcriptase quantitative polymerase chain reaction (RT-qPCR) in liver, spleen, and lung tissue from pigs experimentally infected with the Gram-negative swine specific bacterium *Actinobacillus pleuropneumoniae*, as well as from pigs experimentally infected with the Gram-positive bacterium *Staphylococcus aureus*. Our results show that: 1) SAA1 may be a pseudogene in pigs; 2) we were able to detect two previously uncharacterized SAA transcripts, namely SAA2 and SAA4, of which the SAA2 transcript is primarily induced in the liver during acute infection and presumably contributes to circulating SAA in pigs; 3) Porcine SAA3 transcription is induced both hepatically and extrahepatically during acute infection, and may be correlated to local organ affection; 4) Hepatic transcription of SAA4 is markedly induced in pigs infected with *A. pleuropneumoniae*, but only weakly in pigs infected with *S. aureus*. These results for the first time establish the infection response patterns of the four porcine SAA genes which will be of importance for the use of the pig as a model for human inflammatory responses, e.g. within sepsis, cancer, and obesity research.

## Introduction

Serum amyloid A (SAA) is a small apolipoprotein and a major positive acute phase protein in most vertebrate species [1]. In man, it has consistently been found to be one of the most highly and quickly induced acute phase proteins, with increases in serum concentrations sometimes reaching 1000-fold [2]. During inflammation, SAA becomes the predominant apolipoprotein of high density lipoprotein (HDL), thereby replacing apolipoprotein A1 (APOA1) [3]. The biological function of SAA is debated [4], [5], however the magnitude of hepatic transcription and synthesis of SAA during the early phase of acute inflammation [6], [7], and its high degree of evolutionary conservation across species [8], imply the function of SAA to be remarkably important.

The current SAA nomenclature is based on SAA genes in humans and mice [9]. The human SAA gene cluster comprises four gene loci (i.e. SAA1, SAA2, SAA3P, SAA4), which are organized basically in the same way as the orthologous murine SAA gene loci (i.e. saa1, saa2, saa3, saa4, designated as recommended by Sipe [9]), pointing to a common evolutionary origin of the entire SAA gene cluster [8], [9], [10]. Yet, human SAA1, SAA2, and SAA3P genes are more related to each other than each gene is related to its murine orthologous counterpart, and vice versa [8], indicating subsequent species-specific homogenization of the SAA genes by concerted evolution [8], [11]. Orthologous SAA genes have thus not necessarily retained similar functions, which underlines the need for biological assessment along with sequence analysis. Within each species, the SAA1 and SAA2 genes are highly homologous, while SAA3 is less so [12], [13], and SAA4 is the most divergent member of the SAA family [14], [15]. All genes share the same four-exon three-intron structure [16].

During the acute phase response, human and murine SAA1 and SAA2 gene expression is up-regulated mainly in the liver [10], [17]. Murine saa3 is induced in a wide range of tissues [17], [18], while in contrast, no *in vivo* transcription of human SAA3P has been reported [13], [19]. SAA4 is expressed both in liver tissue and extrahepatically in humans [14], [20], while murine saa4 expression has been found in liver tissue only [15]. SAA4 is not affected to the same extent as SAA1 by inflammation, and is thus not considered an acute phase isoform in neither humans nor mice [14], [21], [22]. SAA1, SAA2, and SAA3 isoforms have been recognized both at transcriptional and protein level in many other species apart from humans and mice [1], [23].

Despite the general lack of immunoassays capable of differentiating between SAA isoforms, analytical methods based on the unique alkaline isoelectric point of SAA3 have shown that in several species SAA3 is the main isoform of local, inflamed tissue, as e.g. in joints in horses [24] and dogs [25], and in bovine udders [26]. In a recent study, mass spectrometry was used to demonstrate the local production of mouse saa3 in adipose tissue of obese mice [27]; interestingly, the locally produced saa3 in this case was not seen to influence the total SAA serum concentration.

As a general rule, SAA3 is thus typically found in both hepatic and non-hepatic tissues during an acute-phase response, while SAA1 and SAA2 are preferentially expressed in the liver. This tissue-specific differential regulation of isoforms during the acute phase response is a unique feature of SAA compared with other acute phase proteins.

As in other species, porcine SAA gene expression is highly induced, both hepatically and extrahepatically, during an acute phase response [28], [29], and elevated levels of circulating SAA protein have been reported as well [30], [31]. Only a single porcine SAA gene transcript has been characterized. This was published by Chang et al. [32] as porcine SAA2 (GenBank accession number DQ367410.1, National Center for Biotechnology Information (NCBI)). Subsequently, others have noted that the transcript shares characteristics of SAA3, including the alkaline peptide sequence motif S-F-L-K in the N-terminal part of the protein [33], in common with the T-F-L-K peptide motif of equine, bovine and ovine SAA3 [34]. Accordingly, this transcript (DQ367410.1 [32]) is named SAA3 throughout the present paper. Porcine SAA3 has been detected in a wide range of tissues, including liver [28], [32], [33], mammary tissue [35], salivary gland, diaphragmatic muscle, lung [33], lymph node, spleen, tonsils, and blood leukocytes [28]. The lack of evidence of expression of other porcine SAA isoforms, together with the physiochemical properties of circulating pig SAA protein [36], has led to the suggestion that SAA3 is the dominant form of circulating SAA in the pig [33], [36]. This would make the pig unique, as SAA3 has not been detected in the circulating pool of any species examined to date [24], [25], [26], [27], [34], as opposed to SAA1, SAA2, and SAA4 [21], [27], [37].

In the present study, the relative expression of porcine SAA1, SAA2, SAA3, and SAA4 genes in liver, spleen, and lung tissue from pigs experimentally infected with the Gram-negative swine-specific pathogen *Actinobacillus pleuropneumoniae* (AP), as well as from pigs experimentally infected with a porcine strain of the Gram-positive pathogen *Staphylococcus aureus* (SA) was studied. In addition to the previously characterized porcine SAA3 transcript [32], we identified two previously uncharacterized porcine SAA transcripts, namely SAA2 and SAA4, while no transcript corresponding to the porcine SAA1 gene was detected. Both infections induced increased hepatic expression of SAA2, SAA3, and SAA4 as well as pulmonary and splenic expression of SAA3.

## Materials and Methods

### Ethics Statement

All animal procedures were conducted according to protocols approved by the Danish Animal Experiments Inspectorate (Licence Numbers 2001/561-350 and 2008/561−1465 for AP and SA infection studies, respectively).

### Primer Design

**Figure 1:**
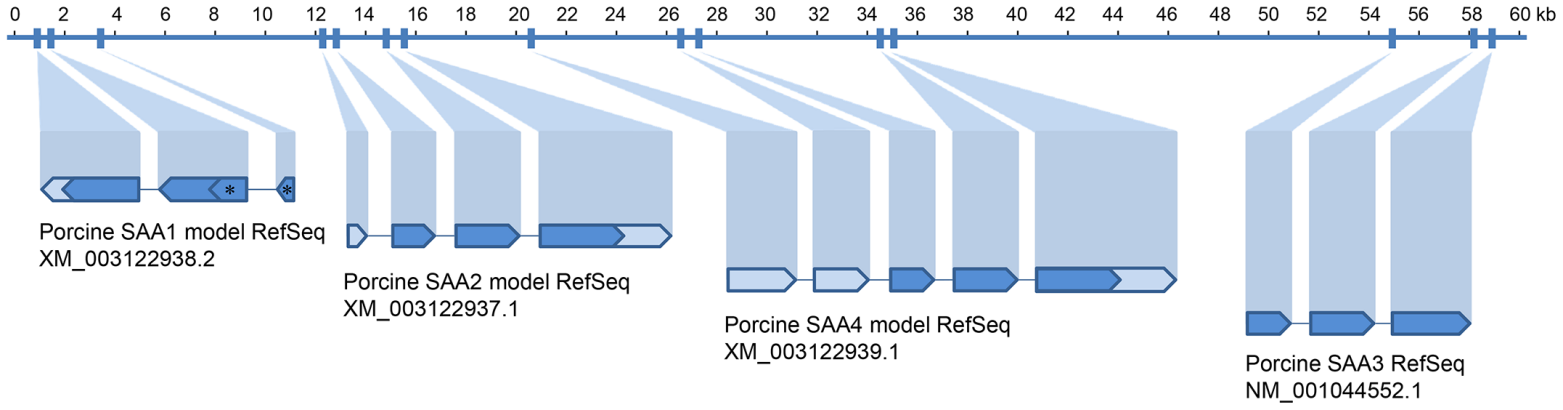
Porcine SAA gene cluster organization. The top line indicates a 60(CU633234.2) onto which the four SAA mRNA reference sequences (RefSeqs) from GenBank, NCBI, are positioned. Arrows show reading frame direction. Light blue is untranslated regions (UTRs). Dark blue is coding domains. Accession numbers are given below the RefSeqs. The SAA3 mRNA RefSeq derives directly from DQ367410.1, published by Chang et al. [32] as SAA2. The other RefSeqs (i.e. the model RefSeqs) derive from NCBI’s automated mRNA prediction methods based on genomic DNA (CU633234.2). *Upstream part of the SAA1 model RefSeq conflicts with the conserved structure of SAA genes in general, and this transcript was not detected in the present study. See discussion in the text.

Specific primer pairs were designed for the putative porcine gene transcripts based on four porcine SAA mRNA reference sequences (RefSeqs) [38] obtained from GenBank, NCBI (accession numbers are given in Figure 1). The SAA3 mRNA RefSeq was derived directly from the porcine SAA cDNA sequence published by Chang et al. [13] (accession number DQ367410.1), while the model mRNA RefSeqs (i.e. SAA1, SAA2, SAA4) had been defined by NCBI’s automated mRNA prediction method GNOMON [39] based on the porcine genome assembly version 10.2 (accession number GCA_000003025.4). All RefSeqs were located within the same genome contig (CU633234.2) (Figure 1).

Primers were designed within the coding domains of the sequences. Due to the extremely high degree of homology between the porcine SAA genes in the downstream part (exons 3 and 4) of the coding domain (Figure S1), it was not always possible to make transcript-specific reverse primers. Forward primers were thus designed to anneal within the upstream 94 bp of the coding domain, i.e. the part of the coding domain with most sequence variation across the four porcine SAA mRNA RefSeqs (see Figures 1 and S1). All primers were designed using Primer3, v 0.4.0 (http://frodo.wi.mit.edu/). Primers were synthesized at Sigma-Aldrich. The primers are listed in Table 1. Geneious v 6.1.2 (Biomatters, http://www.geneious.com) was used as sequence alignment tool.

**Table 1:** Porcine SAA isoform specific primers.

Forward primers		Reverse primers		Ampliconlength (bp)	qPCRefficiency
***Targeting the SAA1 RefSeq***					
** SAA1-F1:**	*TGTCACAGTTTTCTGTTCACCTG*	**A-SAA-R1:**	*CCTTTGGGCAGCATCATAGT*	158	*NA*
** SAA1-F2:**	*TCCTTTTTCCGTGTGCTTTC*	**A-SAA-R1:**	*CCTTTGGGCAGCATCATAGT*	132	1.00
** SAA1-F2:**	*TCCTTTTTCCGTGTGCTTTC*	**A-SAA-R2:**	*GGACATTCTCTCTGGCATCG*	187	*NA*
***Targeting the SAA2 RefSeq***					
** SAA2-F1:**	*GCTGGGAGTCCACAGTCAGT*	**A-SAA-R1:**	*CCTTTGGGCAGCATCATAGT*	157	0.94
***Targeting the SAA3 RefSeq***					
** SAA3-F2:**	*CTCAAGGAAGCTGGTCAAGG*	**A-SAA-R2:**	*GGACATTCTCTCTGGCATCG*	178	0.93
***Targeting the SAA4 RefSeq***					
** SAA4-F1:**	*GGCTTCGGACTTGTGGAGAG*	**SAA4-R1:**	*GATGATTTTTGCTGCCCAGA*	136	*NA*
** SAA4-F2:**	*GTGATGGGAGTCAGCAGTGA*	**SAA4-R2:**	*CTTTGGGCAGCCTCGTAGT*	158	0.95
***Reference gene***					
** TBP-F:**	*ACGTTCGGTTTAGGTTGCAG*	**TBP-R:**	*CAGGAACGCTCTGGAGTTCT*	96	1.02

NA: not analyzed by qPCR (PCR product however confirmed by sequencing).

### *Actinobacillus Pleuropneumoniae* Pig Infection

Experimental design and procedures have been described in detail by Skovgaard et al. [28], [29]. Briefly, three clinically healthy 8–10 weeks old castrates of Danish landrace/Yorkshire/Duroc crossbreeds from a specific pathogen free (SPF) herd were inoculated intranasally with AP serotype 5b, a highly pathogenic porcine bacteria causing severe acute necrotizing pleuropneumonia. The pigs were killed 14–18 h after inoculation and necropsied immediately. Three non-inoculated clinically healthy pigs from the same herd served as control pigs. SAA protein concentration was measured in serum sampled immediately prior to inoculation as well as prior to euthanasia for verification of initiated acute phase response [28]. Tissue samples of approximately 500 mg were obtained from liver, lung, and spleen during necropsy. All samples were snap frozen in liquid nitrogen and stored at –80°C until RNA extraction.

### *Staphylococcus Aureus* Pig Infection

Details of experimental design and procedures are described in Jensen et al. [40] and Leifsson et al. [41]. Briefly, three clinically healthy 8-week-old female SPF Danish landrace/Yorkshire crossbred pigs with a body weight (BW) of 20–25 kg were inoculated intravenously with a bolus of 10^8^ CFU/kg BW of a porcine pathogenic strain of SA. After 12 h, the pigs received a second bolus equal to the first. After 24 h, the pigs were killed, and post mortem examination were immediately done. Three control pigs from the same herd were sham-infected intravenously with sterile saline and managed in parallel with the infected pigs. SAA protein concentration was measured in blood sampled at 0 h (before inoculation) and 24 h PI.

Tissue samples from liver, lungs, and spleen were collected during necropsy and stored in an RNA preserving solution (40 mL 0.5 M EDTA, 25 mL 1 M sodium citrate, 700 g ammonium sulfate, 935 mL sterile distilled water, adjusted to pH 5.2 using H_2_SO_4_). The samples were stored for 24 h at 5°C and thereafter at –20°C until RNA extraction.

### SAA Immunoassay

SAA serum protein analyses were performed on samples from all AP-infected pigs and one AP control pig, and on all SA-infected and SA control pigs, except that one SA control pig was not sampled at 24 h PI.

A commercially available sandwich enzyme-linked immunosorbent assay (ELISA) (Phase SAA assay, Tridelta Development Ltd.) was used for determination of SAA. This assay is based on anti-human monoclonal antibodies in a sandwich set-up, as originally described by McDonald et al. [42]. Samples were tested according to the manufacturer’s instructions, except that the lowest dilution was 1∶20 to increase signal intensity. The detection limit of the assay was 31.25 µg/mL (porcine SAA equivalents). All samples including standards were determined in duplicate. Sample values were calculated from the curve fitted to the standard.

### RNA Extraction, cDNA Conversion

Total RNA was extracted from homogenized tissue samples using RNeasy Kit (Qiagen) (liver and lung samples), and RNeasy lipid midi tissue kit, (Qiagen) (spleen samples). All RNA samples were treated with RNase-free DNase Set (Qiagen), and thereafter kept at –80°C. Quantity, purity and RNA integrity of total RNA were assessed prior to cDNA synthesis with a NanoDrop 1000 Spectrophotometer (Thermo Scientific), and an Agilent 2100 Bioanalyzer (Agilent Technologies). The average (± SD) RNA integrity number (RIN) was 8.2 (±0.3) for SA samples, and 8.5 (±0.2) for AP samples, implying applicable quality of the RNA for qPCR analysis [43]. Extracted RNA was converted to cDNA by reverse transcription of 500 ng total RNA by QuantiTECT Reverse Transcription Kit (Qiagen), using a mix of random primers and oligo-dT.

### qPCR

Quantitative PCR was performed using the RotorGene Q platform (Qiagen). qPCR reaction volumes of 25 µL were prepared using SYBR Green JumpStart Taq ReadyMix (Sigma), 3 mM MgCl_2_, diluted cDNA (×6 or ×8), and gene-specific primers (300 nM). Reactions were performed with two technical replicates as previously described [44], and melting curves were generated after each run to confirm a single PCR product. PCR efficiency for each primer assay was assigned from two standard curves constructed from two separate dilution series. In assays targeting SAA1, SAA2, and SAA3 dilution series were made from pooled equal amounts of all cDNA samples. In the SAA4 assay dilution series were made out of pooled equal amounts of cDNA from liver samples only, as we found that SAA4 was undetectable in lungs and spleen (see Results section).

GenEx5 software (MultiD) was used for efficiency correction of data for each primer assay, and averaging of technical replicates before reference gene normalization. The reference gene TATA box binding protein (TBP) had previously been found highly stable across liver, lung, and spleen tissue in a related SA pig model study [45], and in own studies of AP-infected lung tissue (unpublished data). Based on this, TBP was used as reference gene for normalization of all samples in the present study.

**Figure 2:**
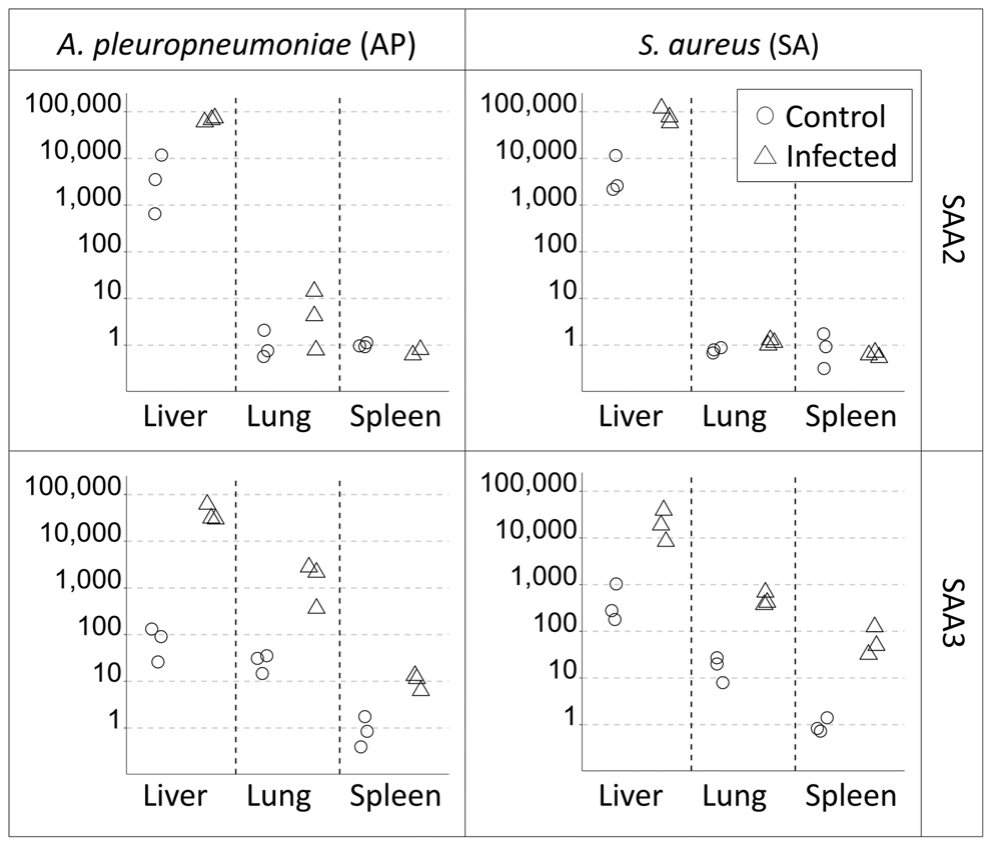
Relative gene expression levels of SAA2 and SAA3 in different tissues for each infection experiment. All samples (*n* = 3 in each group) are shown relative to control spleen tissue, which is set to a mean expression of 1. Please note that Y axis is log10 scale.

For each infection study, gene expression data was presented relative to the mean gene expression of control pigs for each tissue separately (Table 2), as well as relative to mean gene expression of control spleen tissue (Figure 2).

**Table 2:** Gene expression changes in response to infection.

		SAA2		SAA3		SAA4	
		Mean	[Range]	Mean	[Range]	Mean	[Range]
***A. pleuropneumoniae***							
**Liver**	**Infected**	12.77	[11.55–13.78]	499.53	[368.02–754.22]	8.59	[8.29–8.76]
	**Control**	1	[0.12–2.21]	1	[0.32–1.08]	1	[0.53–1.93]
**Lung**	**Infected**	5.7	[0.70–12.62]	67.25	[13.64–106.12]		
	**Control**	1	[0.51–1.83]	1	[0.54–1.31]		
**Spleen**	**Infected**	0.71*	[0.61–0.81]	10.37	[6.25–13.49]		
	**Control**	1	[0.92–1.11]	1	[0.39–1.76]		
***S. aureus***							
**Liver**	**Infected**	16.25	[11.03–23.05]	45.00	[17.11–77.98]	2.14	[1.22–2.82]
	**Control**	1	[0.40–2.12]	1	[0.34–2.11]	1	[0.78–1.13]
**Lung**	**Infected**	1.54	[1.34–1.77]	27.74	[19.94–40.26]		
	**Control**	1	[0.88–1.12]	1	[0.46–1.42]		
**Spleen**	**Infected**	0.63	[0.52–0.72]	67.40	[33.83–119.43]		
	**Control**	1	[0.31–1.75]	1	[0.72–1.44]		

Mean and range of gene expression fold change of SAA2, SAA3, and SAA4 within liver, lung, and spleen tissues of infected pigs (n = 3) compared with control pigs (n = 3; mean gene expression for each isoform set to 1 for each tissue) from the two separate infection experiments. SAA4 was below detection limit in lung and spleen tissue.

* SAA2 was below detection limit in the spleen of one of the AP-infected pigs, and the mean thus represents only two pigs.

### Sequencing

Products from qPCR were used as templates for nucleotide sequencing using the qPCR primers (Table 1).

The templates were purified using MinElute PCR Purification Kit (Qiagen), and concentrations were measured on NanoPhotometer (Implen). PCR was performed using per one reaction: 1 µL BigDye Terminator mastermix, 1.5 µL 5×Sequencing Buffer (BigDye Terminator v3.1 Cycle Sequencing Kit, Applied Biosystems), 1 µL appropriate primer (stock concentration 5 µM), 1–6.5 µL of template cDNA (to attain ∼80 ng of PCR product per sequence reaction), and sterile de-ionized water up to a total volume of 10 µL. PCR was performed on a T3 Thermocycler (Biometra) by 25 consecutive cycles of the following conditions: 96°C for 30 s, 50°C for 15 s, and 60°C for 4 min. PCR products were precipitated by adding 25 µL of 96% ethanol and 1 µL of 3 M sodium acetate (pH 5.2) per reaction and centrifuged for 30 minutes at 15,000×*g*. Precipitated products were washed three times in 70% ethanol, vacuum-dried and resuspended in 10 µL formamide each (Hi-Di Formamide, Applied Biosystems) and finally transferred to a source plate (MicroAmp Optical 96-Well Reaction Plate, Applied Biosystems) to be sequenced. Sanger sequencing was performed using Genetic Analyser 3130 (Applied Biosystems), according to the manufacturer’s manual.

## Results

### Naming of the Four Putative Porcine SAA Genes

The porcine genome (assembly version 10.2) comprised four porcine SAA gene loci closely positioned in a 60 kb region on the porcine chromosome 2 (Figure 1). Following the recommendations by Sipe [9] that SAA isoform nomenclature should be based on the organization of SAA genes in humans and mice [46], [47], we named the four porcine gene loci within the SAA gene cluster 3′-SAA1-5′/5′-SAA2-3′/5′-SAA4-3′/5′-SAA3-3′, respectively (Figure 1).

### Expression Patterns of Porcine SAA Isoforms in Liver, Lung, and Spleen Tissue

Sequencing confirmed identity of obtained PCR products with their target mRNA RefSeqs for SAA2, SAA3, and SAA4 assays. In contrast, no SAA1 transcript was detected. The PCR products obtained from the three different assays used to target the putative SAA1 transcript (Table 1) turned out to be 100% identical to SAA2 DNA sequence, while only 97% identical to the SAA1 mRNA model RefSeq.

A substantially increased expression of SAA2 in the liver of infected pigs compared with control pigs was found in both infection experiments (Table 2). The AP-infected pigs further showed a mean increased relative expression of SAA2 in lung tissue, although with a wide range, while the change in SAA2 expression was negligible in lung tissue from the SA experiment (Table 2). SAA2 regulation was negligible in spleen tissue in both infection experiments (Table 2).

Increased expression of SAA3 was found in liver, lung, and spleen tissue of infected pigs compared with control pigs in both infection experiments (Table 2). Spleen tissue showed the smallest difference in relative expression of all three tissues in the AP experiment while in the SA experiment spleen tissue, in contrast, showed the largest difference in expression, followed by liver, then lung tissue, suggesting differential tissue-specific SAA3 induction between the two infection experiments (Table 2).

Hepatic SAA4 transcription was on average 8.6-fold increased in AP-infected pigs compared to controls, however only slightly increased in SA-infected pigs compared to controls (Table 2). SAA4 transcription was below detection level in lung and spleen tissues.

Generally, while the liver showed the highest expression of both SAA2 and SAA3, compared to lung and spleen, the difference was much bigger for SAA2 than for SAA3 which was more evenly expressed across tissues (Figure 2). This pattern of tissue expression was similar in infected and control pigs.

### Clinical Observations and Gross Pathology

Signs of severe clinical disease developed in all infected pigs from the two infection experiments [28], [40], [41]. The control pigs showed no signs of clinical illness ante mortem, neither any gross lesions post mortem, in either of the two infection experiments.

### SAA Protein in Serum

All infected pigs had increased SAA protein concentrations in serum at the time of killing, indicating an on-going acute phase response (Table 3). The SA-infected pigs had more than three times as high SAA protein concentrations compared with the AP-infected pigs at the time of killing. SAA protein concentrations in the SA control pigs were slightly above detection limit at both sample time points, while the AP control pig, as well as preinoculation concentrations of the infected pigs, all were below detection limit (Table 3).

**Table 3:** SAA serum protein concentrations.

		Before Inoculation		Prior to Euthanization	
		Mean	[Range]	Mean	[Range]
***A. pleuropneumoniae***	Infected (*n* = 3)	≤31.3	NA	136.2	[96.6–197.5]
	Control (*n* = 1*)	≤31.3	NA	≤31.3	NA
***S. aureus***	Infected (*n* = 3)	≤31.3	NA	461.7	[424.0–483.5]
	Control (*n* = 3*)	47.6	[≤31.3–80.4]	41.5	[41.4–41.5]

Mean SAA protein concentration was measured in serum of control pigs and infected pigs from before inoculation (0 h) and prior to euthanization (14–18 h for AP, or 24 h for SA). Detection limit was 31.3 µg/mL. Unit is µg/mL.

* *N* = 3, except serum from only one of the three AP control pigs was analyzed, and only two of the three SA control pigs were sampled at time-point 24 h.

## Discussion

The high degree of sequence homology between SAA isoforms has historically made isoform identification and differentiation difficult, and the search for porcine SAA isoforms has been no exception [36]. However, we established a number of isoform-specific PCR assays and could demonstrate that porcine SAA2 and SAA4 transcription during acute infection is induced within liver tissue, while SAA3 transcription is induced not only in liver tissue, but also in lung and spleen tissue. Also importantly, no transcript corresponding to the SAA1 gene was detected. We used material from two separate infection studies, namely AP experimental infection and SA experimental infection, with markedly different levels of circulating SAA at time of killing (Table 3).

The porcine SAA gene cluster is organized in the same way as both human and murine SAA gene clusters. The similarities between human, murine, and porcine SAA gene clusters thus support the concept of evolution proposed by several authors [8], [10], [48] that the basic organization of the four gene loci within the SAA gene cluster stems back from before divergence of the species.

In accordance with SAA2 in other species [1], [23], we found regulation of the SAA2 transcript mainly within the liver. This could indicate that porcine SAA2 has retained a function which overall is similar to what is found in other species. Knowing that AP infection causes severe acute necrotizing pleuropneumonia, the observed increase in SAA2 expression in lung tissue from AP-infected pigs (Table 2) suggests that severe cases of local organ damage may be accompanied by an increase in local SAA2 expression. Increased extrahepatic expression of SAA2 has been shown in several chronic inflammatory conditions in humans [17], but to our knowledge not in relation to acute inflammation.

SAA3 was clearly induced in all three tissues suggesting that SAA3 is the major extrahepatically inducible SAA isoform in pigs, just as in a number of other animal species, including mouse [18], [49], rat [50], hamster [51], rabbit [52], cow, sheep, and horse [34].

During AP infection, spleen tissue had the lowest increase in relative expression of SAA3 of all three tissues, while the opposite was the case in the SA infection experiment. Although both experimental infections led to highly severe infectious states with clear systemic affection, it is a likely assumption that the mononuclear phagocytic system within the spleen was more severely challenged in the SA experiment, where bacteria were inoculated directly into the blood compartment, compared to the AP experiment, in which the aerogenous route of inoculation inevitably must have delayed and minimized entry of bacteria to the blood stream. Also in spontaneous cases AP does not seem to possess the same ability to cause bacteremia [53] as is highly characteristic of SA [54]. If expression of porcine SAA3 mRNA in spleen tissue reflects the degree of splenic affection or damage, SAA3 could be a potential marker of tissue-specific involvement of acute inflammation in pigs. Differential responses of SAA genes are known from studies in mice, where injection of both lipopolysaccharide (LPS) and casein were equally able to induce hepatic SAA1 and SAA2, while only LPS, but not casein, induced hepatic as well as extrahepatic SAA3 expression, showing that induction of SAA is not just an all-or-none response [18].

SAA4 transcription was induced as well, although most markedly in the AP experiment (8.6-fold). The level of expression in AP-infected pigs seems prominent in the light of the current perception of SAA4 as a constitutive isoform, and prompts a need for more thorough analysis of the role of this gene in porcine inflammation.

The four-exon three-intron organization is a highly conserved feature of SAA genes [16], of which the upstream first exon is part of the untranslated region (UTR) [17]. Whereas the current version of the SAA3 mRNA RefSeq contains coding domains only, and thus lacks a first exon, the differing number of exons in the SAA1 and SAA4 model mRNA Refseqs (Figure 1) is likely due to inaccuracy of the mRNA prediction methods employed. The porcine SAA1 mRNA model RefSeq shows a different organization of the region normally expected to lie as a separate exon 2 (Figure 1, asterisks), and when translated, this part of the sequence clearly differs from the other porcine isoforms (Figure S1, gray italics), as well as from all other known SAA isoforms across species (not shown). The unusual N-terminal sequence along with the unusual exon organization, and our inability to detect it, makes it probable that this predicted transcript (XM_003122938.2) does not exist. Therefore, when relying on the current version of the porcine genome, it is not unlikely that SAA1 could be a pseudogene in pigs like the murine SAA-ps gene which also contains only exons three and four [12]. The murine SAA-ps locus is, however, positioned differently relative to the other SAA loci (i.e. upstream of SAA4), so the two genes are unlikely to be evolutionary orthologs. It should be noted that the current version of the porcine genome contig (CU633234.2) is stated only as a working draft sequence, therefore, in order to fully address the complete organization of the porcine SAA1 locus, an updated sequencing of the genomic region in question seems to be needed.

It is not known which isoform(s) make(s) up the circulating SAA in pigs, and the SAA protein assay used in this study may not reflect all SAA isoforms equally. Second, gene expression levels can only be compared for each gene individually. Third, possible differences in mRNA and protein kinetics are not accounted for. However our discovery of porcine SAA2 transcriptional induction during acute infection in itself questions the conclusion made by others [33] that SAA3 is the major circulating isoform in pigs. Also, the finding that the serum concentration of SAA in SA-infected pigs was more than three times that of the AP-infected pigs (Table 3), taken together with the fact that hepatic SAA3 was more highly induced in the AP experiment than in the SA experiment (Table 2), suggests that circulatory SAA does not solely consist of SAA3. Although this result may to some degree be explained by the earlier sample time-point of AP-infected pigs, it nevertheless favors the assumption that SAA2 contributes to circulating acute phase SAA in the pig, just as in other species [1], [23]. Differentiation of serum SAA isoforms at protein level may be accomplished in the future by mass spectrometry, of which the potential diagnostic possibilities within the veterinary field remain to be elucidated.

In summary, the porcine SAA superfamily consists of four SAA gene loci, namely SAA1, SAA2, SAA3, and SAA4, of which SAA2, SAA3, and SAA4 are expressed and differentially induced during acute infection.

These results are of importance for the understanding of the biology of SAA and for gaining further insight into the inflammatory response of the pig.

## Supporting Information

Figure S1 **Porcine SAA mRNA RefSeq alignments.** Only coding domains are shown. The inferred protein sequence is given below each RefSeq. The ‘divergent’ part of the putative SAA1 protein is indicated in gray italics (see Discussion). Asterisk indicates stop codon.
(PDF)
