# Supplementary material for: Organization and Biology of the Porcine Serum Amyloid A (SAA) Gene Cluster: Isoform Specific Responses to Bacterial Infection
Source: PLoS One. 2013 Oct 11;8(10):e76695. doi: 10.1371/journal.pone.0076695 (PMC3795699; doi:10.1371/journal.pone.0076695)
Supplement: Figure S1 — Porcine SAA mRNA RefSeq alignments. Only coding domains are shown. The inferred protein sequence is given below each RefSeq. The ‘divergent’ part of the putative SAA1 protein is indicated in gray italics (see Discussion). Asterisk indicates stop codon. (PDF) [file pone.0076695.s001.pdf]

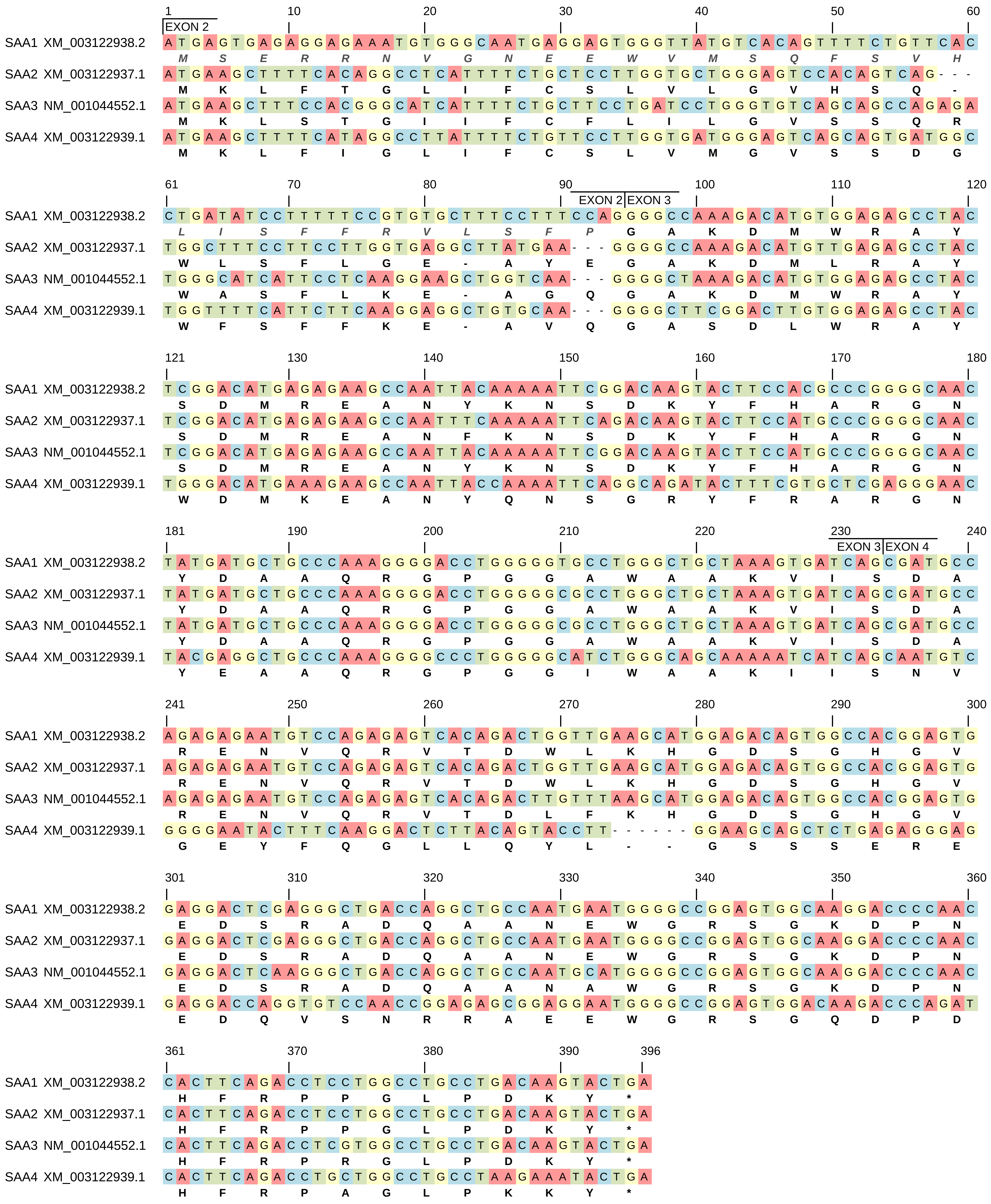
EXON 2
EXON 2 | EXON 3
EXON 3 | EXON 4
SAA1 XM_003122938.2
SAA2 XM_003122937.1
SAA3 NM_001044552.1
SAA4 XM_003122939.1
